# Integration of Artificial Intelligence for Diagnostic Methods in Musculoskeletal Conditions: A Systematic Review

**DOI:** 10.7759/cureus.79391

**Published:** 2025-02-20

**Authors:** Akshanda Dhumale, Sandeep Shinde, Manoj P Ambali, Prakash Patil

**Affiliations:** 1 Musculoskeletal Sciences, Krishna College of Physiotherapy, Krishna Vishwa Vidyapeeth (Deemed to be University), Karad, IND; 2 Anatomy, Krishna College of Physiotherapy, Krishna Vishwa Vidyapeeth (Deemed to be University), Karad, IND; 3 Radiodiagnosis, Krishna Institute of Medical Sciences, Karad, IND

**Keywords:** 3d printing, artificial intelligence, assistive devices and pervasive technology, health data, machine learning, musculoskeletal diseases

## Abstract

Artificial intelligence (AI) is a multi-disciplinary area of research focused on understanding, simulating, and replicating intelligence and cognitive functions by applying computational, mathematical, logical, mechanical, and biological principles and technologies. The concept of AI involves investigating and exploring human intelligence and creating artificial computers that use intelligent algorithms to replicate human intelligence. With the appearance of machine learning (ML), deep learning (DL), and convolutional neural networks (CNNs), the key AI techniques that are particularly effective in capturing feature items and learning, AI has evolved into a powerful approach in image analysis. AI may enable more precise evaluations of musculoskeletal impairments, reducing the likelihood of misdiagnosis and improving treatment outcomes for patients. With improved diagnostic capabilities, physiotherapists can create tailored rehabilitation programs that cater to the specific needs and conditions of individual patients.

This study aimed to explore and evaluate the integration of AI technologies in diagnostic methods to enhance assessment accuracy. A systematic review was conducted from available literature on AI applications in musculoskeletal diagnostics. Available articles from 2015 to 2025 were included in the study. Analysis of current research's trends, advantages, constraints, and gaps was recognized.

This study highlights the promising role of AI technologies in enhancing the accuracy and efficiency of musculoskeletal diagnostics. The integration of AI has the potential to revolutionize diagnostic methods, offering more precise assessments and reducing the likelihood of misdiagnosis. The issue of deploying AI tools for diagnostic purposes needs more attention.

## Introduction and background

The term "artificial intelligence" (AI) refers to a branch of computer science dedicated to enabling computers to replicate human cognitive abilities [1]. Machine learning (ML) is an area of AI that allows computers to learn and recognize patterns in data without explicit programming [2]. ML is a type of AI that uses patient data to automate decision-making and predictions. ML is classified into two types: supervised and unsupervised. Supervised ML is a type of ML in which algorithms provide training data that is analyzed and labeled for classification features. The model is "trained" using this data before being tested with unlabeled data. For example, if the data consists of X-ray images, a radiographer labels them first so that the model can learn how to interpret them. The model is then evaluated with unlabeled data to generate an interpretation result. Unsupervised ML is employed to detect patterns without the need for training. Cluster analysis (grouping data based on patterns of attributes) and association (discovering principles that govern data) are two common types. Healthcare is well-suited for AI, as the complex datasets derived from electronic health records (EHRs) offer challenging classification opportunities. Additionally, improvements in pathways and task automation can help reduce costs [3]. AI is a significant opportunity to improve professional success and performance. Integration of AI in healthcare offers unique opportunities to improve patient care outcomes, decision-making, and professional performance and reduce costs [4]. In supervised algorithms, the categories are set in advance. These classes are established as a finite set determined by humans, meaning that a specific portion of data will be assigned these labels. The goal of the ML algorithm is to discover patterns and develop mathematical models. The models are evaluated based on their ability to predict, considering the variance present in the data [5]. Unsupervised ML eliminates the requirement for human supervision of the model, unlike supervised learning. This sub-category of ML allows a system to operate on particular data without external instruction [6]. AI emerged as a potential tool in the realm of medical imaging, offering potential solutions to improve fracture diagnosis in orthopedic X-rays. By analyzing the characteristics of fractures and comparing them to existing knowledge, AI algorithms can assist in determining the type of fracture and its associated complications [7].

AI-assisted medical diagnosis has been successfully applied in detecting various lung abnormalities, such as pulmonary nodules, cancer, pneumothorax, mediastinal widening, consolidation, pleural effusion, atelectasis, fibrosis, calcification, and even acute respiratory distress syndrome, in X-ray and computed tomography (CT) scans, yielding promising results [8]. In orthopedics, X-ray, CT, and magnetic resonance imaging (MRI) are the most commonly used methods for diagnosing musculoskeletal conditions. These imaging techniques offer minimal errors when detecting micro fractures, occult fractures, or non-displaced fractures, as well as other orthopedic diseases with nonspecific symptoms, including osteoporosis, arthritis, ligament and cartilage injuries, bone deformities, tumors, and bone age evaluation [9]. There are three widely used approaches to assess medical images using deep learning (DL): classification, object detection, and segmentation. Classification is used to identify the appropriate class to which the input image corresponds. Object detection identifies the presence or absence of a specific object and marks its location with a bounding box. Segmentation reports the exact pixel-wide margin of an object [10]. The convolutional neural network (CNN) is a form of neural network that uses the convolution operation as a core element in its design. The CNNs are primarily used for pattern recognition with images, and they utilize their convolutional filters to extract the information from images. The images further categorize the impairment in the image in accordance with the input data [11].

## Review

Methodology

Study Design

The systematic review was carried out following the Preferred Reporting Items for Systematic Reviews and Meta-Analyses (PRISMA) guidelines to guarantee transparency, consistency, and methodological integrity in presenting the review process and results.

Search Strategy

For this systematic review, an extensive search strategy was employed, utilizing multiple academic search engines, including PubMed, Web of Science, Scopus, and Research Gate. These platforms were selected to ensure a comprehensive and robust collection of relevant studies, encompassing a wide range of peer-reviewed articles, conference proceedings, and grey literature, thereby minimizing the risk of publication bias and ensuring the inclusion of the most pertinent and up-to-date evidence available.

Selection criteria

Inclusion Criteria

Studies that focused on integration of AI in musculoskeletal diagnosis were included in the study. Full text articles and articles published in the last 10 years were studied. Experimental and comparative studies that address physical diagnosis with the help of AI in musculoskeletal conditions were included.

Exclusion Criteria

Studies that were unpublished, in abstract form only, lacked peer-reviewed publication, and duplicate articles were excluded from the study.

Data Extraction and Quality Assessment

Data extraction was conducted systematically using a standardized data extraction form designed to capture key study characteristics, methodology, and outcomes. The extracted data included study details like author, year, study design, outcome measures, and key findings. Two independent reviewers performed the data extraction process, and any discrepancies were resolved through discussion or consultation with a third reviewer.

Results

**Figure 1:**
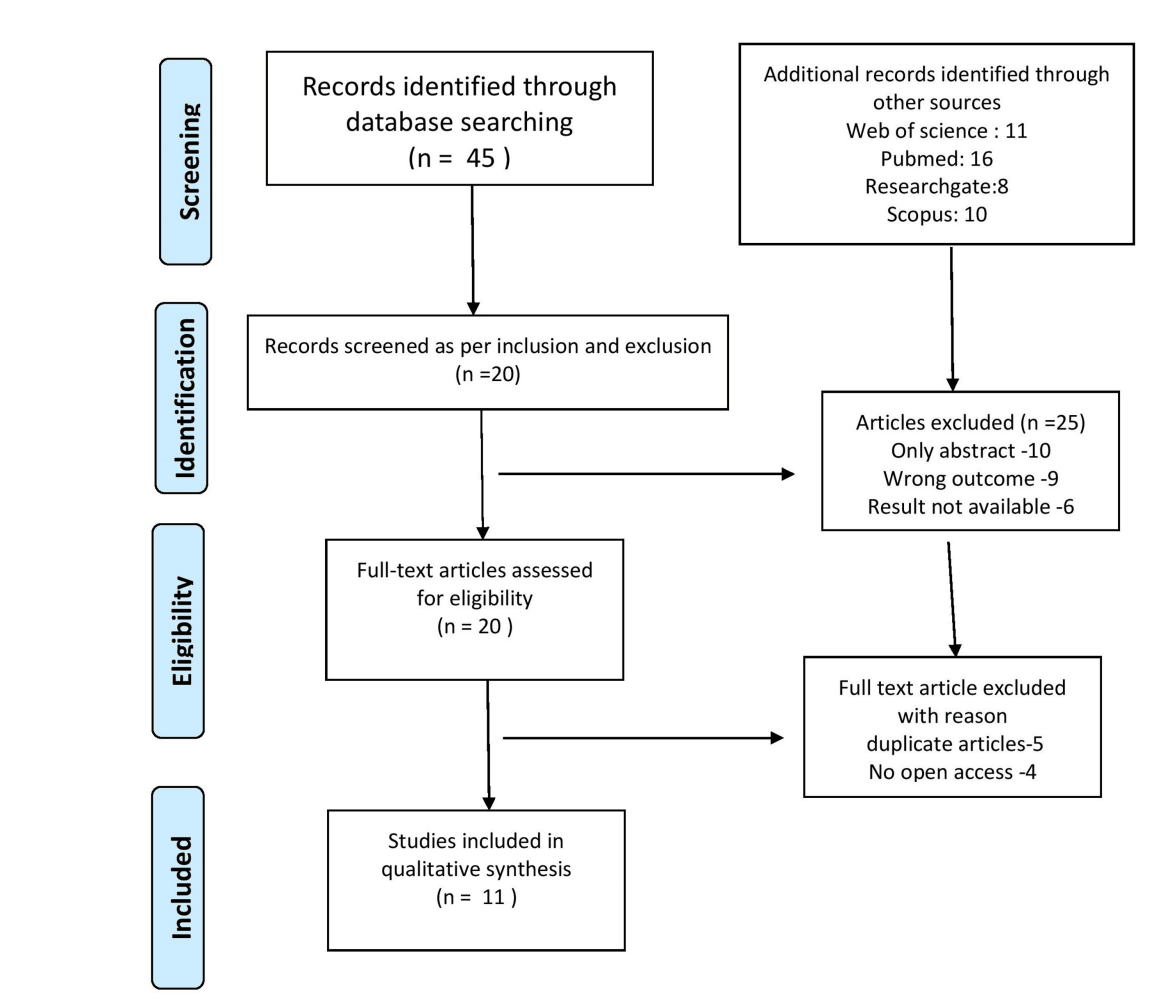
Preferred Reporting Items for Systematic Reviews and Meta-Analyses (PRISMA) flow chart

Out of the 45 articles reviewed, 20 met the criteria for inclusion in the final analysis. Eleven articles were later finalized. Figure 1 shows the PRISMA flow chart. The exclusion of more than half of the articles implies that there were many studies that were either too broad in scope, not rigorous enough in their methods, or did not specifically focus on full text. AI technologies are particularly valuable in physiotherapy for their ability to analyze large datasets quickly and with precision. For example, AI tools can analyze X-rays, MRI scans, or videos of patients performing exercises and assist physiotherapists in making more accurate diagnoses. The use of AI could reduce human error, help identify conditions earlier, and improve the standardization of diagnoses across different clinics and practitioners.

The characteristics of the 11 articles that were studied are presented in Table 1.

**Table 1:** Characteristics of the included articles ACL: anterior cruciate ligament; AI: artificial intelligence; ANN: artificial neural network; AUC: area under curve; BMI: body mass index; CAD: computer-aided diagnosis; CNN: convolutional neural network; CT: computed tomography; MAIA: machine learning assisted image annotation; MRI: magnetic resonance imaging; PRC: precision recall curve; SERA: sepsis early risk assessment; TPF: tibial plateau fractures

Sr. no.	Year	Author	Type of article	Intervention	Outcome	Results	Analysis
1.	2021	Sato et al. [12]	Experimental study	Pytorch 1.3 and Fast.ai 1.0 EfficientNet-B4 model (a pre-trained ImageNet model)	Calculated the accuracy, sensitivity, specificity, F-value, and ROC curve and measured the AUC.	The accuracy, sensitivity, specificity, F-value, and AUC were 96.1%, 95.2%, 96.9%, 0.961, and 0.99, respectively, with Gradient-Weighted Class Activation Mapping generating the correct diagnostic framework. In the controlled study, the diagnostic accuracy of the residents was enhanced with the use of the CAD system.	This study innovated a deep learning-based CAD system for detecting the hip fractures observed in X-ray images, demonstrating that the system significantly improved diagnostic accuracy among less experienced residents, with the model achieving high performance (96.1% accuracy, 0.99 AUC).
2.	2021	Liu et al. [13]	Comparative study	RetinaNet	The detection accuracy rate was used to check the accuracy.	The AI algorithm demonstrated a detection accuracy of 0.91 in identifying TPF, matching the performance of orthopedic physicians. (0.92 ± 0.03). The AI completed the analysis in an average of 0.56 seconds, which is 16 times quicker than the time taken by humans (8.44 ± 3.26 seconds).	The research used a dataset of 542 X-ray images of TPFs to train the AI algorithm, RetinaNet, for detecting fractures. The performance of the AI was measured using detection accuracy and analysis time.
3.	2016	Parisi et al. [14]	Comparative review	Self-organizing map and supervised multilayer perceptron neural networks.	Dempster-Shafer theory-based classifier.	The study used biomechanical data from 38 patients with late-stage knee osteoarthritis and 38 healthy volunteers. The results showed the classification accuracy of each method, with a focus on their ability to accurately distinguish between healthy and osteoarthritic knee function.	This study compares the performance of four different classifiers - Cardiff classifier (Dempster-Shafter theory-based), ANN, support vector machine, and Lagrangian support vector machines - to differentiate between healthy and osteoarthritic knee function using gait data from 38 patients with knee osteoarthritis and 38 healthy volunteers. The research involves advanced machine learning techniques and biomechanical data analysis to assess knee function and the suitability of these classifiers for clinical use.
4.	2022	Chen et al. [15]	Experimental study	MAIA automatic deep learning software for medical imaging analyses. MRI scans for ACL diagnosis.	The CNN model of MAIA was used based on EfficientNet-B0, pretrained with ImageNet.	The AI models achieved high diagnostic accuracy, with the first model correctly differentiating between torn and intact ACL images with an accuracy of 99.46% and a final ACL tear diagnosis accuracy of 96%. The second model identified torn ACL images with 99.43% accuracy, while the third model distinguished between torn and intact ACLs with 96.91% accuracy, outperforming less experienced clinicians across all metrics.	This study shows that AI, using convolutional neural networks, can effectively diagnose ACL tears from MRI images with high accuracy. The AI models outperformed less experienced clinicians in diagnosing ACL tears, offering a promising tool for clinical decision-making.
5.	2021	Goh et al. [16]	Comparative study	SERA algorithm	ROC curve	The SERA algorithm evaluates sepsis risk by using clinical notes and structured data for each patient consultation, with two linked components: one diagnosing sepsis at the time of consultation and another predicting sepsis risk for the next four to 48 hours, while avoiding bias from critical care-confirmed cases.	Sepsis is a major cause of hospital deaths. Early detection and diagnosis of sepsis are crucial for reducing mortality, but it is challenging because its symptoms often overlap with those of other, less serious conditions. They have developed the SERA algorithm, an artificial intelligence system that utilizes both structured data and unstructured clinical notes to predict and diagnose sepsis. They assess this algorithm with independent clinical notes and achieve strong predictive accuracy, detecting sepsis 12 hours before its onset (AUC 0.94, sensitivity 0.87 and specificity 0.87). We evaluate the SERA algorithm against physician predictions and highlight its potential to improve early sepsis detection by up to 32% and decrease false positives by up to 17%. Mining unstructured clinical notes has been shown to enhance the algorithm’s accuracy, outperforming the use of only clinical measures for early warning 12 to 48 hours before sepsis onset.
6.	2022	Pongsakonpruttikul et al.[17]	Experimental study	YOLOv3 tiny	PRC AUC	The AI model using YOLOv3 tiny could detect and classify normal and osteoarthritic knees on plain knee joint radiographs with 85% accuracy and 81% mean average precision. The second AI model for classifying severity achieved a total accuracy of 86.7% and mean average precision of 70.6%.	This study developed AI models using CNNs to assist in the detection and classification of knee osteoarthritis from radiographs, achieving high accuracy in distinguishing between normal and osteoarthritic knees as well as classifying the severity of osteoarthritis. With accuracies of 85% for detection and 86.7% for severity classification, these AI models show great potential as diagnostic aids, helping radiologists and orthopedists efficiently identify osteoarthritis stages and guide treatment decisions.
7.	2017	Xue et al. [18]	Comparative study	CNN model consisting of 16 layers. VGG-16 layer network.	Diagnosis accuracy rate	The CNN model achieved a sensitivity of 95.0% and specificity of 90.7%, with a diagnostic accuracy rate of 92.8%, comparable to that of chief physicians. The model performed better with more experienced physicians, and its diagnostic decisions aligned with the features identified by human doctors, such as joint space narrowing and osteophytes in X-ray images.	This study demonstrates that a deep CNN trained on 420 hip X-ray images achieved high sensitivity (95.0%) and specificity (90.7%), with a diagnostic accuracy of 92.8%, showing its potential to perform at a level comparable to experienced physicians in diagnosing hip osteoarthritis.
8.	2015	Al-Helo et al.[19]	Comparative study	K-means and neural network	Gradient vector flow snake	Our overall accuracy was 98% for K-means and an average of 93.2% for neural network testing set. K-means showed high specificity of 99.1% and acceptable sensitivity of 87.1%.	This study presents a fully automated CAD system for diagnosing vertebra wedge compression fractures from CT images, achieving high diagnostic accuracy of 93.2% with neural networks and 98% with K-means clustering. The system integrates into clinical workflows, offering promising results for future clinical applications after further validation.
9.	2017	Jamaludin et al.[20]	Comparative study	CNN	Class average accuracy	The detection system achieved 95.6% accuracy in terms of disc detection and labeling. The model is able to produce predictions of multiple pathological gradings that consistently matched those of the radiologist. The model identifies “Evidence Hotspots” that are the voxels that most contribute to the degradation scores.	This study demonstrates the development of an automated system for grading lumbar intervertebral discs and vertebral bodies from MRIs, achieving 95.6% accuracy in disc detection and labeling, with predictions matching those of radiologists. The system improves the objectivity and speed of radiological analysis, offering potential benefits for clinical diagnoses and large-scale research on back pain.
10	2019	Shah et al. [21]	Experimental study	Double-echo steady-state MRI series were included in our analysis.	Articular cartilage thickness measured via machine learning	In a study of 3910 MRIs, the average femoral articular cartilage thickness was 2.34 mm, with significant regional variations; distal medial and lateral cartilage were thinner than posterior cartilage. Multivariate analysis revealed that male sex and higher BMI were associated with thicker articular cartilage, while female sex and lower BMI correlated with thinner articular cartilage (all P < 0.001).	This study utilizes machine learning to automate the segmentation and measurement of articular cartilage thickness in healthy knees using MRI, providing normative data and identifying the influence of demographic variables like sex and BMI on articular cartilage thickness variation.
11	2021	Zdolsek et al. [22]	Experimental study	Deep neural networks, specifically the ResNet50, VGG19, and InceptionV3 models	Diagnostic accuracy, sensitivity, specificity, and AUC	In the automated pathway, ResNet50 demonstrated the highest diagnostic accuracy, with an average of 91% (SD 1.3), outperforming VGG19 at 83% (SD 1.6) and InceptionV3 at 89% (SD 2.5). The accuracy levels for the intervention pathway were 94% (SD 2.0) for ResNet50, 92% (SD 2.7) for VGG19, and 93% (SD 3.7) for InceptionV3. In terms of sensitivity and specificity, ResNet50 outperformed the other networks, achieving a mean AUC (area under the curve) of 0.94 (SD 0.01), surpassing the accuracy of clinical diagnostics.	This study developed and tested deep neural networks, particularly ResNet50, to accurately distinguish atypical femoral fractures from normal femur fractures on radiographs, with manual image orientation improvements further enhancing diagnostic accuracy, surpassing clinical diagnostics.

Risk of Bias Assessment

The risk of bias for the included studies was assessed to ensure the reliability of the findings. To ensure the reliability and validity of the findings, the methodological quality of the included studies was assessed using appropriate tools. As mentioned in Table 2, the Risk of Bias in Non-randomized Studies - of Interventions (ROBINS-1) was used.

**Table 2:** Risk of Bias in Non-randomized Studies - of Interventions (ROBINS-1)

Study	Bias due to confounding	Bias in selection of participants	Bias in classification of interventions	Bias due to deviations from intended interventions	Bias due to missing data	Bias in measurement of outcomes	Bias in selection of the reported result
Sato et al. [12]	Moderate risk	Low risk	Low risk	Low risk	Low risk	Moderate risk	Moderate risk
Liu et al. [13]	Moderate risk	Moderate risk	Low risk	Low risk	Low risk	Moderate risk	Moderate risk
Parisi et al. [14]	Moderate Risk	High risk	Low risk	Low risk	Low risk	Moderate risk	Low risk
Chen et al. [15]	Moderate risk	Low risk	Low risk	Low risk	Low risk	Low risk	Moderate risk
Goh et al. [16]	Moderate risk	Moderate risk	Low risk	Low risk	Low risk	Moderate risk	Moderate risk
Pongsakonpruttikul et al.[17]	Moderate risk	Moderate risk	Low risk	Low risk	Low risk	Low risk	Moderate risk
Xue et al. [18]	Moderate risk	Low risk	Low risk	Low risk	Low risk	Moderate risk	Moderate risk
Al-Helo et al.[19]	Moderate risk	High risk	Low risk	Low risk	Low risk	Low risk	Moderate risk
Jamaludin et al.[20]	Moderate risk	Low risk	Low risk	Low risk	Low risk	Moderate risk	Moderate risk
Shah et al. [21]	Moderate risk	Moderate risk	Low risk	Not applicable	Low risk	Moderate risk	Moderate risk
Zdolsek al. [22]	Moderate risk	Moderate risk	Low risk	Low risk	Low risk	Moderate risk	Moderate risk

Discussion

The implementation of AI into musculoskeletal diagnosis represents a promising advancement, offering the capability to enhance assessment accuracy and improve treatment outcomes. As demonstrated in the growing body of literature, AI technologies are seeing growing implementation in clinical settings to detect abnormalities and predict treatment efficacy.

Chen et al., in their study, provided insights into the current trends and advanced imaging modalities to help diagnose even the smallest impairment in X-rays, MRI, or CTs [23]. Frizziero et al., in their study, explained the significance of 3D printing in diagnoses of fractures. The application of additive manufacturing in creating 3D-printed bone models has shown to be an effective tool for enhancing preoperative planning in orthopedic surgeries, as it enables precise anatomical reproduction [24]. Cipolletta et al. stated that ML algorithms demonstrate significant potential in enhancing the analysis of advanced imaging techniques like fluorescence optical imaging (FOI), primarily in relation to inflammation in rheumatic diseases. By identifying and ranking key imaging features, these algorithms can improve diagnostic accuracy, facilitating the early detection and effective management of various rheumatic conditions [25]. Overgaard et al. designed an AI model competent of identifying osteophytes and scoring them using a validated semiquantitative system for ultrasound (US) imaging. The agreement between the expert reader and the AI algorithm was found to be slightly higher than the agreement levels previously observed between experts when evaluating osteophytes in US images of hand joints, showing potential for use in diagnosing and assessing hand osteoarthritis [26]. Bhat et al. concluded AI and ML could transform musculoskeletal medicine by increasing diagnostic accuracy, tailoring treatments, and advancing rehabilitation. However, challenges such as data privacy, AI validation, and the complexity of musculoskeletal conditions must be addressed for successful clinical integration [27]. Droppelmann et al. suggested that AI models evaluated in this study revealed impressive accuracy in detecting musculoskeletal pathologies of the upper extremity, but challenges such as data complexity, variability in image interpretation, and incorporation into clinical practice need to be addressed [28]. Egereonu et al. explored that integration of an expert system for diagnosing musculoskeletal diseases shows significant promise in improving diagnostic accuracy, streamlining healthcare processes, and enhancing patient outcomes. However, further advancements in AI-driven diagnostic systems, including real-time application and continuous learning, are necessary to fully realize their potential in clinical settings and improve overall healthcare delivery [29]. Shin et al.'s recent advancements in US imaging technology have been associated with enhanced accuracy in diagnosis. Computer-aided detection (CADe)/computer-aided diagnosis (CADx) systems offer quantitative analysis and provide an additional perspective, aiding radiologists in making accurate and consistent image assessments efficiently. DL-based US CADe/CADx systems are considered the next generation of AI-powered radiology, as trained models can be reliable and generalizable, benefiting from the availability of big data in digitalized radiology [30]. Yu et al., in their study, used the SONIMAGE HS1 PLUS color Doppler US diagnostic instrument (Konica Minolta, Inc., Tokyo, Japan). The study concluded that musculoskeletal US combined with an AI algorithm for pain rehabilitation of scapulohumeral periarthritis is a novel and simple method with high diagnostic efficiency [31]. Gyftopoulos et al. studied that radiomics is an emerging field in medicine that is based on the extraction of diverse quantitative characteristics from images and the use of these characteristics for data mining and pattern identification. These data can then be used with other patient information to better characterize and predict disease progress based on an ML algorithm [32]. Vitiello et al. concluded that radiology, an essential aspect of diagnosing musculoskeletal tumors, has seen the introduction of AI-driven algorithms to detect and distinguish between benign and malignant lesions across different imaging techniques. Histopathology, essential in diagnosis and treatment decisions, is enhanced by AI, which accelerates tumor classification through pattern recognition [33]. Kijowski et al.'s research on DL methods has shown promising results in detecting fractures on CT as well as identifying osseous metastatic disease through CT and nuclear medicine [34]. Teresa et al. underscore that the clinical applications, MRI image reconstruction, joint localization, level of severity determination, knee osteoarthritis prediction, arthritis distinction, and disease-specific joint regions identification based on ML algorithms are of great importance. Neural networks are used for accurate localization of the disease [35].

Clinical Implications

AI-powered models, particularly approaches in DL, including CNN, have demonstrated high diagnostic accuracy and precision in detecting and classifying musculoskeletal conditions like fractures, osteoarthritis, and cartilage damage. By automating and standardizing image analysis, these AI systems can reduce human error, expedite diagnosis, and provide consistent interpretations across different clinicians and settings. Various algorithms including SERA, YOLOv3 tiny, MLP, ResNet50, VGG19 have been proven to improve the accuracy of the diagnosis. AI models have been shown to enhance the detection of knee osteoarthritis, hip fractures, and vertebral compression fractures, often outperforming less experienced clinicians. These tools can act as valuable decision support systems, guiding treatment decisions and improving patient care outcomes. Furthermore, AI's potential to handle large datasets efficiently makes it an essential asset in clinical environments, reducing workload, optimizing resource allocation, and enabling more timely and accurate diagnoses.

Future Scope

AI has the ability to revolutionize the field of medicine, by optimizing outcomes through data-driven decision-making. In particular, AI has the potential to enhance the accuracy and efficiency of diagnostic processes by analyzing complex medical data, such as imaging, genetic information, and patient history, in ways that surpass human capabilities. With advancements in DL, AI systems can continuously improve their diagnostic accuracy by learning from vast datasets, allowing them to detect diseases at earlier stages, even before symptoms become apparent. Moreover, AI-powered tools could help reduce human error, minimize diagnostic delays, and provide personalized treatment recommendations tailored to individual patients. With refined algorithms through DL and ML, AI is increasingly being utilized to manage complex datasets, identify variable associations, and improve the value and accuracy of clinical outcomes. These advancements enable more personalized patient care, such as preemptive risk profiling for adverse events in surgeries and the ability to assess the impact of various interventions on patient satisfaction and clinical results.

Limitations

Despite the advancements, the broad implementation of AI in clinical settings encounters notable obstacles, such as high hardware expenses, inconsistencies in data management, and the necessity for validation across various healthcare systems. To train AI systems, large, appropriately labeled databases are needed, which are expensive to build. As AI technologies continue to evolve, they hold immense promise for transforming orthopedic care and improving clinical decision-making, though realizing their full potential will demand overcoming both technical and practical barriers.

## Conclusions

This study concludes that integration of AI in the diagnosis of musculoskeletal conditions, particularly through DL models, has shown promising results in improving diagnostic accuracy, reducing human error, and providing timely, consistent interpretations. These AI systems have exhibited their ability to effectively aid in identifying and classifying musculoskeletal conditions like osteoarthritis, fractures, ligament injuries and cartilage damage, thus assisting clinicians in reaching more informed conclusions and improving patient care. Future advancements in AI for musculoskeletal diagnosis should focus on improving model generalization across diverse patient demographics, enhancing interpretability, integrating seamlessly with clinical workflows, enabling real-time decision support, incorporating predictive analytics for personalized care, and addressing regulatory and ethical concerns.

## References

[1] Gitto S, Serpi F, Albano D, Risoleo G, Fusco S, Messina C, Sconfienza LM (2024). AI applications in musculoskeletal imaging: a narrative review. Eur Radiol Exp.

[2] Erickson BJ, Korfiatis P, Akkus Z, Kline TL (2017). Machine learning for medical imaging. Radiographics.

[3] Tack C (2019). Artificial intelligence and machine learning | applications in musculoskeletal physiotherapy. Musculoskelet Sci Pract.

[4] Abuzaid MM, Elshami W, Hegazy F, Aboelnasr EA, Tekin HO (2022). The impact of artificial Intelligence (AI) in physiotherapy practice: a study of physiotherapist willingness and readiness. J Hunan Univ Nat Sci.

[5] Nasteski V (2017). An overview of the supervised machine learning methods. Horizons.

[6] Naeem S, Ali A, Anam S, Ahmed MM (2023). An unsupervised machine learning algorithms: comprehensive review. Int J Comput Digital Syst.

[7] Sharma S (2023). Artificial intelligence for fracture diagnosis in orthopedic X-rays: current developments and future potential. SICOT J.

[8] Liu P, Zhang J, Liu S (2024). Application of artificial intelligence technology in the field of orthopedics: a narrative review. Artif Intell Rev.

[9] Pinto A, Berritto D, Russo A (2018). Traumatic fractures in adults: missed diagnosis on plain radiographs in the emergency department. Acta Biomed.

[10] Ko S, Pareek A, Ro DH, Lu Y, Camp CL, Martin RK, Krych AJ (2022). Artificial intelligence in orthopedics: three strategies for deep learning with orthopedic specific imaging. Knee Surg Sports Traumatol Arthrosc.

[11] Innocenti B, Radyul Y, Bori E (2022). The use of artificial intelligence in orthopedics: applications and limitations of machine learning in diagnosis and prediction. Appl Sci.

[12] Sato Y, Takegami Y, Asamoto T (2021). Artificial intelligence improves the accuracy of residents in the diagnosis of hip fractures: a multicenter study. BMC Musculoskelet Disord.

[13] Liu PR, Zhang JY, Xue MD (2021). Artificial intelligence to diagnose tibial plateau fractures: an intelligent assistant for orthopedic physicians. Curr Med Sci.

[14] Parisi L, Biggs PR, Whatling GM, Holt CA (2015). A novel comparison of artificial intelligence methods for diagnosing knee osteoarthritis. XXV ISB Congress.

[15] Chen KH, Yang CY, Wang HY, Ma HL, Lee OK (2022). Artificial intelligence-assisted diagnosis of anterior cruciate ligament tears from magnetic resonance images: algorithm development and validation study. JMIR AI.

[16] Goh KH, Wang L, Yeow AY, Poh H, Li K, Yeow JJ, Tan GY (2021). Artificial intelligence in sepsis early prediction and diagnosis using unstructured data in healthcare. Nat Commun.

[17] Pongsakonpruttikul N, Angthong C, Kittichai V (2022). Artificial intelligence assistance in radiographic detection and classification of knee osteoarthritis and its severity: a cross-sectional diagnostic study. Eur Rev Med Pharmacol Sci.

[18] Xue Y, Zhang R, Deng Y, Chen K, Jiang T (2017). A preliminary examination of the diagnostic value of deep learning in hip osteoarthritis. PLoS One.

[19] Al-Helo S, Alomari RS, Ghosh S (2013). Compression fracture diagnosis in lumbar: a clinical CAD system. Int J Comput Assist Radiol Surg.

[20] Jamaludin A, Lootus M, Kadir T (2017). ISSLS Prize in Bioengineering Science 2017: automation of reading of radiological features from magnetic resonance images (MRIs) of the lumbar spine without human intervention is comparable with an expert radiologist. Eur Spine J.

[21] Shah RF, Martinez AM, Pedoia V, Majumdar S, Vail TP, Bini SA (2019). Variation in the thickness of knee cartilage. The use of a novel machine learning algorithm for cartilage segmentation of magnetic resonance images. J Arthroplasty.

[22] Zdolsek G, Chen Y, Bögl HP, Wang C, Woisetschläger M, Schilcher J (2021). Deep neural networks with promising diagnostic accuracy for the classification of atypical femoral fractures. Acta Orthop.

[23] Chen W, Zhang Y, Wang X, Liu J (2024). Comparative analysis of imaging modalities for diagnosing musculoskeletal disorders. J Innov Med Res.

[24] Frizziero L, Liverani A, Donnici G (2019). New methodology for diagnosis of orthopedic diseases through additive manufacturing models. Symmetry.

[25] Cipolletta E, Fiorentino MC, Vreju FA, Moccia S, Filippucci E (2024). Artificial intelligence in rheumatology and musculoskeletal diseases. Front Med (Lausanne).

[26] Overgaard BS, Christensen AB, Terslev L, Savarimuthu TR, Just SA (2024). Artificial intelligence model for segmentation and severity scoring of osteophytes in hand osteoarthritis on ultrasound images. Front Med (Lausanne).

[27] Bhat R, Santhanam V, Sekar K, Gite S, Naik N, Talyshinskii A (2025). Chapter 2 - identification and classification of musculoskeletal conditions using artificial intelligence and machine learning. Diagnosing Musculoskeletal Conditions Using Artificial Intelligence and Machine Learning to Aid Interpretation of Clinical Imaging.

[28] Droppelmann G, Rodríguez C, Jorquera C, Feijoo F (2024). Artificial intelligence in diagnosing upper limb musculoskeletal disorders: a systematic review and meta-analysis of diagnostic tests. EFORT Open Rev.

[29] Egereonu SK, Ekedebe N, Otuonye AI, Etus C, Amadi EC, Egereonu UU (2024). Development of an expert system for diagnosing musculoskeletal disease. Int J Intell Inf Syst.

[30] Shin Y, Yang J, Lee YH, Kim S (2021). Artificial intelligence in musculoskeletal ultrasound imaging. Ultrasonography.

[31] Yu L, Li Y, Wang XF, Zhang ZQ (2023). Analysis of the value of artificial intelligence combined with musculoskeletal ultrasound in the differential diagnosis of pain rehabilitation of scapulohumeral periarthritis. Medicine (Baltimore).

[32] Gyftopoulos S, Lin D, Knoll F, Doshi AM, Rodrigues TC, Recht MP (2019). Artificial intelligence in musculoskeletal imaging: current status and future directions. AJR Am J Roentgenol.

[33] Vitiello R, Ziranu A, Maccauro G (2024). Artificial intelligence in musculoskeletal oncology. Artificial Intelligence in Orthopaedic Surgery Made Easy.

[34] Kijowski R, Liu F, Caliva F, Pedoia V (2020). Deep learning for lesion detection, progression, and prediction of musculoskeletal disease. J Magn Reson Imaging.

[35] Teresa M (2023). Assessing musculoskeletal abnormalities with deep learning. J Widya Medika Junior.

